# Comparison of the performance of an amplicon sequencing assay based on Oxford Nanopore technology to real-time PCR assays for detecting bacterial biodefense pathogens

**DOI:** 10.1186/s12864-020-6557-5

**Published:** 2020-02-17

**Authors:** Robert Player, Kathleen Verratti, Andrea Staab, Christopher Bradburne, Sarah Grady, Bruce Goodwin, Shanmuga Sozhamannan

**Affiliations:** 1 The Johns Hopkins University Applied Physics Laboratory, Laurel, MD USA; 2 Naval Surface Warfare Center, Dahlgren, VA USA; 3 Defense Biological Product Assurance Office, JPEO-CBRND Enabling Biotechnologies (JPEO-CBRND-EB), 110 Thomas Johnson Drive, Frederick, MD 21702 USA; 4 Logistics Management Institute, Tysons, VA USA

**Keywords:** Biodefense, Biodetection, Biosurveillance, Oxford Nanopore sequencing, Real-time PCR, High throughput PCR assay, LoD, Singleplex, Multiplex

## Abstract

**Background:**

The state-of-the-art in nucleic acid based biodetection continues to be polymerase chain reaction (PCR), and many real-time PCR assays targeting biodefense pathogens for biosurveillance are in widespread use. These assays are predominantly singleplex; i.e. one assay tests for the presence of one target, found in a single organism, one sample at a time. Due to the intrinsic limitations of such tests, there exists a critical need for high-throughput multiplex assays to reduce the time and cost incurred when screening multiple targets, in multiple pathogens, and in multiple samples. Such assays allow users to make an actionable call while maximizing the utility of the small volumes of test samples. Unfortunately, current multiplex real-time PCR assays are limited in the number of targets that can be probed simultaneously due to the availability of fluorescence channels in real-time PCR instruments.

**Results:**

To address this gap, we developed a pipeline in which the amplicons produced by a 14-plex end-point PCR assay using spiked samples were subsequently sequenced using Nanopore technology. We used bar codes to sequence multiple samples simultaneously, leading to the generation and subsequent analysis of sequence data resulting from a short sequencing run time (< 10 min). We compared the limits of detection (LoD) of real-time PCR assays to Oxford Nanopore Technologies (ONT)-based amplicon sequencing and estimated the sample-to-answer time needed for this approach. Overall, LoDs determined from the first 10 min of sequencing data were at least one to two orders of magnitude lower than real-time PCR. Given enough time, the amplicon sequencing approach is approximately 100 times more sensitive than real-time PCR, with detection of amplicon specific reads even at the lowest tested spiking concentration (around 2.5–50 Colony Forming Units (CFU)/ml).

**Conclusions:**

Based on these results, we propose amplicon sequencing assay as a viable alternative to replace the current real-time PCR based singleplex assays for higher throughput biodefense applications. We note, however, that targeted amplicon specific reads were not detectable even at the highest tested spike concentrations (2.5 X 10^4^–5.0 X10^5^ CFU/ml) without an initial amplification step, indicating that PCR is still necessary when utilizing this protocol.

## Background

Nucleic acid sequencing-based bioagent detection applications have recently gained momentum in the microbial diagnostics and biosurveillance arenas [1, 2]. Historically, however, the field of human genetics has led the way in advancing sequence-based diagnostics, following the advent of Next Generation Sequencing (NGS) technologies just over a decade ago [3]. While there are Laboratory Developed Tests (LDTs) and Food and Drug Administration (FDA) cleared amplicon sequencing-based cancer and cardiac panel assays in use in clinical practice [4, 5], not many Commercial Off-The-Shelf (COTS) products for microbial detection or diagnostics are currently available. Polymerase Chain Reaction (PCR) assays, developed in many formats and on multiple platforms, continue to be the gold standard in nucleic acid based-microbial detection and diagnostics due its ease of use, widespread instrument availability, and relatively low cost. However, to continue improving assay detection performance, the microbial community must keep pace with the changing landscape of sequencing technologies.

The United States Department of Defense (DoD) and other Government agencies engaged in biosurveillance have substantial interest in detecting pathogens that could potentially be used in a bioterror attack. As such, they have invested heavily in the development and deployment of biological detection technologies [6, 7]. Many diagnostic and biosurveillance strategies utilize PCR-based amplification to detect pathogen-specific genomic fragments, or antibody-based detection of pathogen-specific antigenic proteins or whole pathogens [8, 9]. PCR, while sensitive, can (i) be confounded by inhibitors, (ii) give false negative and false positive results due to target sequence variations and near-neighbor perfect target matches, or (iii) yield varying degrees of amplification efficiencies, impacting limit of detection (LoD) measurements. Due to these shortfalls, there is a need for orthogonal, confirmatory tests such as highly sensitive sequencing to provide adequate confidence in the initial PCR positive results prior to implementation of protective measures.

Recent advances in NGS technologies offer improved sensitivity for microbial detection/diagnosis compared to other detection strategies, both in clinical and environmental point of need/point of care settings [10]. Indeed, high throughput amplicon sequencing assays have previously been used to detect several pathogens [11–13]. This concept is made even more attractive by the availability of Third Generation Sequencers (TGS) such as the handheld MinION devices from Oxford Nanopore Technologies (ONT), which do not require the substantial infrastructure or hardware capital investment of other benchtop sequencing technologies. To demonstrate the utility of TGS in a field environment, the biosurveillance community needs a use case that demonstrates the successful deployment of these devices in field-forward environments or at the point of care. These scenarios are typically constrained by operational and logistical requirements (e.g. power and cold-chain management), and require systems that demand minimal technical expertise and provide user-friendly post-sequencing analysis tools. In this study, we have tested a use case in which a field laboratory technician would utilize a multiplex PCR assay with follow-on amplicon sequencing by the MinION as a replacement assay for multiple singleplex PCR assays. We present data that support the idea that TGS can handle multiplexed, high-throughput detection of critical pathogens in a given sample at a substantial reduction in overall cost and time as compared to current real-time PCR based approaches.

## Results

### Rationale for experimental approach

Current biosurveillance strategies predominantly employ singleplex real-time PCR assays that interrogate a single target sequence in a given pathogen. Actionable calls on any suspected pathogen in a sample are made based on positive amplification of more than one target found in that pathogen. For example, in order to determine that pathogenic *Bacillus anthracis* is present in a sample, one has to detect at least three separate targets; one on the chromosome and two virulence associated sequences found on separate plasmids. Similar procedures are used for other biothreat agents. In addition, the number of samples that can be screened is laboratory-dependent and depends on the capacity for high-throughput sample processing (manual versus automated sample preparation, available PCR instrumentation etc). In this study, we aimed to develop a multiplexed, high-throughput, amplicon sequencing assay utilizing the ONT MinION device. We addressed a specific scenario where aerosols are collected on filters, which were subsequently screened for the presence of a set of biodefense related pathogens. In our experimental approach, different attenuated and further inactivated (chemical or irradiation) pathogens of known concentrations were spiked into three different matrices: cocktail buffer (CB), clean filter (CF), and dirty filter (DF). DFs were generated with buffer containing background organisms collected from aerosol sampling made over a period of time from different locations (Table 1 for sample groups, Table 2 for spike concentrations).

**Table 1:** Multiplex strategy for 513 total spiked samples

Set	Organism(s)	Strain	Dilution Steps	Matrices	Replicates	Total Barcodes	Flowcell#
1	*Francisella tularensis*	239	5	3	3	45	1
	*Francisella tularensis*	240	5	3	3	45	2
	*Francisella tularensis*	241	5	3	3	45	3
	*Yersinia pestis*	113	5	3	3	45	4
	*Yersinia pestis*	114	5	3	3	45	5
	*Burkholderia mallei*	164	5	3	3	45	6
	*Burkholderia pseudomallei*	197	5	3	3	45	7
	*Bacillus anthracis*	708-gi	5	3	3	45	8
	*Bacillus anthracis*	708-live	5	3	3	45	9
2	All agents (known, amplified)	all 9	4	3	3	36	10
	All agents (blinded, amplified)	all 9	4	3	3	36	11
	All agents (known, unamplified)	all 9	4	3	3	36	12

Set 1 is composed of 405 samples, split into 9 single agent cocktails with 8 unique agents among them. Set 2 is composed of 108 samples, split into 3 combined agent cocktails containing all 8 unique agents (708-gi, not 708-live). In this study, cocktails refers to samples suspended in buffer solution

**Table 2:** Target Concentration (CFU/ml) of spiked materials for *Set 1* and *Set 2*

Set	Step	Strain								
		239	240	241	113	114	164	197	708-gi	708-live
1	5	1.25E+ 05	2.50E+ 04	9.53E+ 04	2.50E+ 05	2.50E+ 05	5.00E+ 05	5.00E+ 05	2.50E+ 05	2.50E+ 05
	4	1.25E+ 04	2.50E+ 03	9.53E+ 03	2.50E+ 04	2.50E+ 04	5.00E+ 04	5.00E+ 04	2.50E+ 04	2.50E+ 04
	3	1.25E+ 03	2.50E+ 02	9.53E+ 02	2.50E+ 03	2.50E+ 03	5.00E+ 03	5.00E+ 03	2.50E+ 03	2.50E+ 03
	2	1.25E+ 02	2.50E+ 01	9.53E+ 01	2.50E+ 02	2.50E+ 02	5.00E+ 02	5.00E+ 02	2.50E+ 02	2.50E+ 02
	1	1.30E+ 01	2.50E+ 00	9.53E+ 00	2.50E+ 01	2.50E+ 01	5.00E+ 01	5.00E+ 01	2.50E+ 01	2.50E+ 01
2	5	1.25E+ 05	2.50E+ 04	9.53E+ 04	2.50E+ 05	2.50E+ 05	5.00E+ 05	5.00E+ 05	2.50E+ 05	n/a
	4	1.25E+ 04	2.50E+ 03	9.53E+ 03	2.50E+ 04	2.50E+ 04	5.00E+ 04	5.00E+ 04	2.50E+ 04	n/a
	3	1.25E+ 03	2.50E+ 02	9.53E+ 02	2.50E+ 03	2.50E+ 03	5.00E+ 03	5.00E+ 03	2.50E+ 03	n/a
	2	1.25E+ 02	2.50E+ 01	9.53E+ 01	2.50E+ 02	2.50E+ 02	5.00E+ 02	5.00E+ 02	2.50E+ 02	n/a

Concentrations were selected to ensure consistent detection by real-time PCR (C_t_ < 30) at the highest concentration(s). The “Step” column indicates position within the serial 10 fold dilution sequence. n/a: not applicable

### Limited multiplex PCR and generation of amplicons for sequencing (preparation for set 1)

As an initial test of the performance of the proposed sequencing approach, we performed limited multiplex PCR for each spiked agent. These multiplex reactions consisted of 3 to 4 species-specific assays that targeted different regions of the pathogen genome. Amplification of each target was detected using a different probe fluorophore (Table 3). In Set 1 (a single spiked agent per sample) primers and probes targeting different regions of the pathogen were used in the PCR reaction. For example, for samples containing *B. anthracis*, which requires 3 assays to test positive for identification, three compatible fluorescent probes (FAM, VIC and NED) were used. Similarly, *Yersinia*, *Francisella*, and *Burkholderia* spiked samples were evaluated in 4 plex, 4 plex, and 3 plex format, respectively, using different fluorophores to assess their performance in the same reaction. The number of attenuated strains spiked on to filters varied for each species, depending on which strains contained the target sequences. For example, the engineered *B. anthracis* strain used contained all three target sequences, but two *Yersinia* strains, three *Francisella* strains, and two *Burkholderia* strains had to be used to test all corresponding agent assays. The expected and observed PCR results and the efficiencies of the different assays are presented in Table 4.

**Table 3:** Detailed PCR assay information

Organism	Strain(s)	PCR Assay ID_Number	Molecule Lengths (bp)				Probe Dye /Channel	Quencher	PCR Plex
			Forward Primer	Probe	Reverse Primer	Amplicon			
*B. anthracis*	*708*	PRC_01	29	30	26	110	FAM	QSY (3′)	3
*B. anthracis*	*708*	PRC_04	20	27	20	182	VIC	QSY (3′)	
*B. anthracis*	*708*	PRC_07	21	31	20	96	NED	QSY (3′)	
*Y. pestis*	*113,114*	PRC_09	19	25	22	68	FAM	QSY (3′)	4
*Y. pestis*	*113*	PRC_11	23	30	25	79	VIC	QSY (3′)	
*Y. pestis*	*114*	PRC_14	20	27	22	103	NED	QSY (3′)	
*Y. pestis*	*113,114*	PRC_15	22	26	17	67	CY5	QSY (3′)	
*F. tularensis*	*239,240,241*	PRC_23	30	33	25	135	FAM	QSY (3′)	4
*F. tularensis*	*239*	PRC_28	25	30	24	171	VIC	QSY (3′)	
*F. tularensis*	*240*	PRC_29	30	40	27	119	NED	QSY (3′)	
*F. tularensis*	*241*	PRC_30	33	25	31	126	CY5	QSY (3′)	
*B. mallei*	*164*	PRC_49	24	20	20	100	FAM	QSY (3′)	3
*B. pseudomallei*	*197*	PRC_50	24	27	20	115	VIC	QSY (3′)	
*B. pseudomallei*	*197*	PRC_65	18	23	19	67	NED	QSY (3′)	

Probe (usage count): FAM (4), VIC (4), NED (4), CY5 (2). Organism and strains shown with matching PCR assay(s). Primer and probe lengths also presented with associated real-time PCR channels used for detection

**Table 4:** Summary of limited multiplex real-time PCR results for individual agents

Organism	Strain	*F. tularensis* assays				*Y. pestis* assays				*Burkholderia* assays			*B. anthracis* assays		
*F. tularensis*	239	122	78	TN	TN	–	–	–	–	–	–	–	–	–	–
	240	116	TN	FN	TN	–	–	–	–	–	–	–	–	–	–
	241	48	TN	TN	79	–	–	–	–	–	–	–	–	–	–
*Y. pestis*	113	–	–	–	–	75	76	TN	84	–	–	–	–	–	–
	114	–	–	–	–	85	TN	FN	98	–	–	–	–	–	–
*B. mallei*	164	–	–	–	–	–	–	–	–	148	TN	TN	–	–	–
*B. pseudomallei*	197	–	–	–	–	–	–	–	–	FP	71	71	–	–	–
*B. anthracis*	708-gi	–	–	–	–	–	–	–	–	–	–	–	66	46	46
	708	–	–	–	–	–	–	–	–	–	–	–	33	46	46
PCR Assay Number		**23**	**28**	**29**	**30**	**9**	**11**	**14**	**15**	**49**	**50**	**65**	**01**	**04**	**07**
Probe Dye / Channel		FAM	VIC	NED	CY5	FAM	VIC	NED	CY5	FAM	VIC	NED	FAM	VIC	NED

Values in the cells indicate an observed positive result (C_t_ < 40) where a positive result was expected, and represent a PCR efficiency percentage. A minus sign (−) indicates the assay-organism combination was not tested. Cells containing FN or FP indicate an observed false negative or positive result, respectively. Cells containing TN indicate an observed negative or undetected result (C_t_ ≥ 40) where a positive result was *not* expected

The majority of the assays gave only the expected true positive and true negative results with three exceptions: assay 49 gave a false positive result against *Burkholderia* 197, and assays 29 and 14 gave false negative results against *Francisella* 240 and *Yersinia* 114. The false positive result is somewhat confounding, as the subsequent sequencing results did not produce any corresponding target amplicon read data (see Results - MinION sequencing for details). For.

the false negative results, sequencing analysis revealed corresponding reads in both instances. Neither assay produced an amplification curve or a C_*t*_ value in PCR. As both of these assays used the NED fluorophore, it is possible that the instrument’s detection in this channel was not functioning properly at the time. Subsequent real-time PCR analysis of *Yersinia* 114 and *Francisella* 240 as individual agents interrogated with the 14-plex primer/probe mix showed that both assays performed as expected (see Results – Multiplex real-time PCR of Mixed Agent and Mixed primer/probe Cocktails (prep for Set 2) for details).

**Fig. 1:**
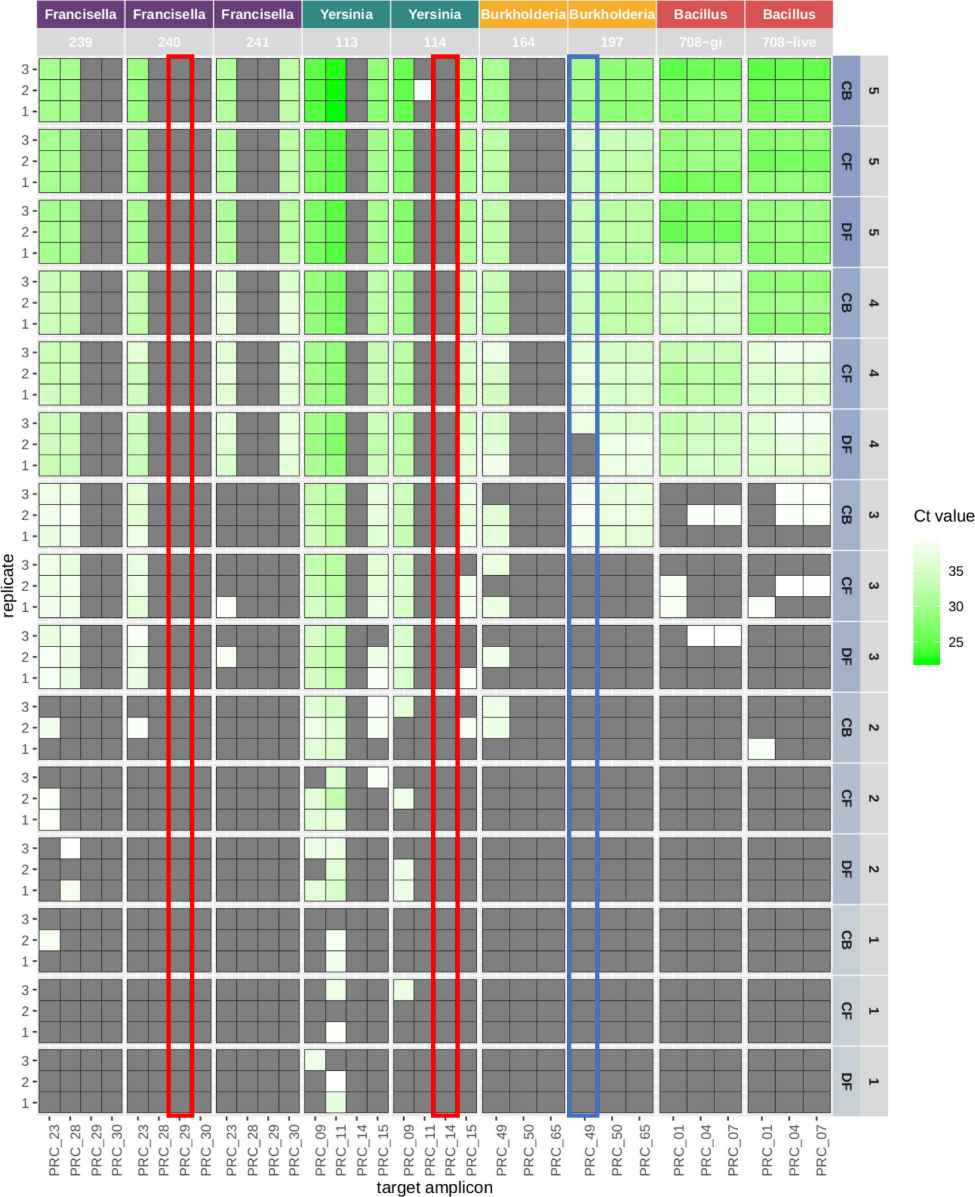
Heat map of C_*t*_ values of limited multiplex real time PCR data. Real time PCR results of set 1expressed as a heat map of C_*t*_ vales as a function of the spiked concentrations of different organisms. The intensity of green color scale represents C_*t*_ values; i.e., dark shades of green indicating lower C_*t*_ values. Grey boxes indicate ‘undetected’ (i.e., >C_*t*_ of 40) by real time PCR. Organism and corresponding strains are indicated across the top, condition and spiking concentrations (CFUs) (10 fold dilution steps 5 through 1) are along the right side (numbers in Table 2), and replicate number along the left side. Conditions are as follows: CB cocktail buffer, CF clean filter, and DF dirty filter. The x-axis indicates assay (or amplicon reference). The red and blue rectangles indicate false negative and positive results, respectively

Further analysis of the real-time PCR data was performed by plotting the C_*t*_ values as a function of the concentration of spiked agent (Fig. 1). As expected, in all cases except for the two false negatives and one false positive, there is a corresponding increase in C_*t*_ values as spiked concentrations decreased. Results in Fig. 1 also revealed that additional fine-tuning of PCR conditions is still necessary to optimize the performance of these assays. At 100% efficiency, PCR assays are expected to show an increase in C_*t*_ value of 3.3 following 10-fold dilutions. Our results show an average shift of 4.2 C_*t*_ value between all sequential 10-fold dilutions for all tested assays in Set 1 samples. The PCR efficiencies varied from 33 to 148% depending on the agent, assay and conditions (Table 4). Similar results were obtained with respect to PCR efficiencies when these assays were performed in a singleplex format (Additional file 2: Table S1).

Additional findings are as follows: 1) there are differences between strains with respect to limits of detection. The most common highest spiking concentration is 2.5 × 10^5^ CFU/ml. The exceptions to this are strains 164 and 197 (both are 5.0 × 10^5^ CFU/ml) and strains 239, 240, and 241 (1.3 × 10^5^, 2.5 × 10^4^, and 9.5 × 10^4^ CFU/ml, respectively). We observed that the differences in detection limits are not commensurate with the differences in spike concentrations. For example, strains 113 and 114 were spiked at about the same CFU/ml as strain 708, yet the LoDs are roughly 2 orders of magnitude lower for strains 113 and 114. 2) For the same strain, there are assay specific differences in their detection limits attributable to copy number differences between chromosome and plasmid. Assays 09 and 11, which detect targets on multi-copy plasmids present in strain 114, for example, have lower LoD values than assay 15, which detects a genomic target. 3) The same assay shows different performance in different strains. For example, the LoD for assay 23 in strain 241 is roughly one order of magnitude lower than strain 240, likely due to a 2 base pair mismatch at the 3′ end of the target amplicon region in the reference genome of strain 241. 4) Potential cross contamination is seen in some cases: assay 11 tested in strain 114 at the highest spike concentration gave a false positive in replicate number two. 5) Species-specific differences are also seen: assays employing vegetative *F. tularensis* and *Y. pestis* cells as input have lower LoDs then those using *B. anthracis* spores. This could be due to differences in DNA extraction efficiencies, as extracting nucleic acids from spores is typically less efficient than extractions from vegetative cells [14, 15]. 6) There appears to be a difference in C_*t*_ values when comparing gamma-irradiation (*gi*) inactivated and live spores of the same organism (compare 708-*gi* to 708-live). This may be attributable to degradation of DNA inside the spores during the gamma irradiation inactivation process, leading to the degradation of the target sequence. 7) There are differences in DNA extraction efficiencies from different matrices. Extraction from the CB matrix appears to be the most efficient, followed by the CF and DF matrices. Overall, these results establish LoD baselines for each assay when tested in different strains, and highlight the inherent differences in sample extraction and PCR efficiencies when performed even in a limited multiplex format. Each of these individual PCR assays was designed and tested independently. These results highlight the need to test all assays moving forward for compatibility in a multiplex format, as well as matching amplification efficiencies as closely as possible.

### Sequencing of set 1 amplicons

**Fig. 2:**
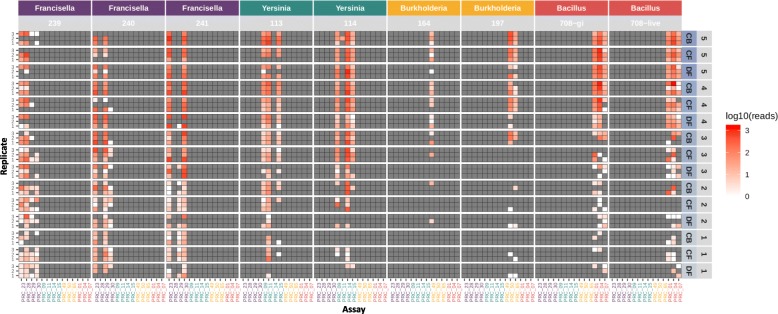
Heat map of sequence read counts from limited multiplex real time PCR reactions (10 min data). Amplicon sequence data represented as a heat map of read counts of set 1 amplicon sequencing on ONT platform (only first 10 min of sequencing data presented). Expected assay results are presented in Table 4. The intensity of red indicates the number of read counts in log10 scale. Organism and corresponding strains are indicated across the top, condition and spiking concentrations (Colony Forming Units) (10 fold dilution steps 5 through 1) are along the right side (numbers in Table 2), and replicate number along the left side. Conditions are as follows: CB cocktail buffer, CF clean filter, and DF dirty filter. The x-axis indicates assay (or amplicon reference)

**Fig. 3:**
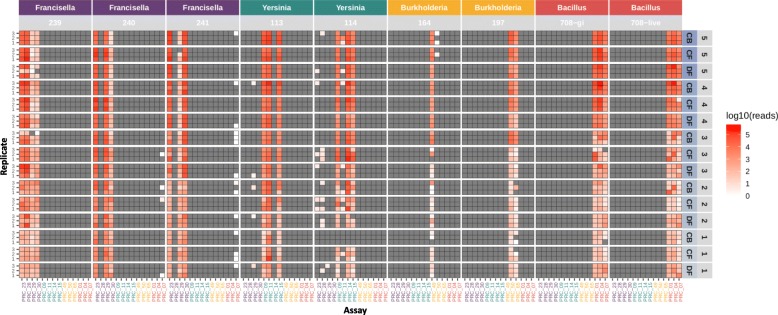
Heat map of sequence read counts from limited multiplex real time PCR reactions (48 h data). Amplicon sequence data represented as a heat map of read counts of set 1 amplicon sequencing on ONT platform (full 48 h of sequencing data presented). Expected assay results are presented in Table 4. The intensity of red indicates the number of read counts in log10 scale. Organism and corresponding strains are indicated across the top, condition and spiking concentrations (CFUs) (10 fold dilution steps 5 through 1) are along the right side (numbers in Table 2), and replicate number along the left side. Conditions are as follows: CB cocktail buffer, CF clean filter, and DF dirty filter

The batch of individually spiked samples (Set 1) contained 9 preparations with 45 samples each (Table 1). Each block of 45 samples was barcoded according to the sequencing library preparation protocol outlined in the Methods section and run on a single R9.5 Nanopore flowcell. The sequence data from two different time points (10 min and 48 h) were processed and analyzed (minutes 1–9 data are presented as an animated gif, Additional file 1: Figure S1). The raw sequence data were base-called, de-multiplexed, and mapped to a BWA database of reference amplicon sequences as described in the Methods section [16]. Only mapped reads with a MAPQ (mapping quality) score ≥ 60 (correlating to at least a 99.9999% probability that the mapping of the read is correct) were considered for these analyses. Read counts as a function of the spiked concentration were plotted as heat maps (Figs. 2 and 3).

### Sequence data for first 10 minutes of sequencing run

After only 10 min of sequencing, a sufficient number of reads were produced to make a conservative positive call on agent presence or absence in the sample at most spiked concentrations (median amplicon mapped read count of 80 for expected positive amplicons). In a majority of the samples, agent specific amplicon reads were detectable even at the lowest concentrations (Fig. 2). Depending on the assay, this represents at least a 1 to 2 order of magnitude improvement in LoD compared to real-time, singleplex PCR alone. Some false positive reads were seen (strains 239, 240 and 241), and are detailed in the following section.

### Sequence data for full 48 hours of sequencing run

A summary and breakdown of read counts for the full 48 h of sequencing data are shown in Table 5, and results of amplicon read mapping are presented in Fig. 3. The number of reads per sample (replicate) after adapter and quality trimming (QC) ranged from 0 to over 4.3 million, with a median of 67,717. The general patterns of true positives and other differences between assays and strains are similar to the results seen in the first 10 min of sequencing data, but here the read counts are much higher (48 h: 10 min median amplicon mapped read count ratio of 4.3:1 as opposed to a ratio of 380:1 considering the median numbers from all samples), allowing correct calls to be made at even the lowest spike concentrations.

**Table 5:** Read count ranges for first 10 min and full 48 h of sequencing data

Run Time	Preprocessing	Sample Count	Group	Read Counts		
				min	max	median
10 Minutes	Adapter + Quality trimmed	405	all samples	0	11,013	178
		405	mapped per sample	0	1697	27
		5670	mapped per assay	0	1502	0
		1129	mapped per assay (zeros removed)	1	1502	80
48 Hours	Adapter + Quality trimmed	405	all samples	0	4,322,566	67,717
		405	mapped per sample	0	579,930	10,803
		5670	mapped per assay	0	472,720	0
		1561	mapped per assay (zeros removed)	1	472,720	346

Mapped in this table refers to amplicon mapped. Note that for the ‘mapped per assay’ group, the median value includes counts for assays that should remain at zero, i.e. are true negatives

False positive read counts were also substantially elevated in the 48 h data (e.g., assay 07 in most samples spiked with *Francisella* 241). The increased read counts collected over 48 h also revealed potential cross contamination of PCR assay products that are not identified in the first 10 min of data (Table 6). For example, strain specific reads from different *Francisella* strains were present in strains not expected to produce those reads, *B. anthracis* reads were present in several *Francisella* samples, *Francisella* reads were present in several *Yersinia* samples, and *Burkholderia* 197 reads were present in several *Burkholderia* 164 samples. We note that these false positive, cross contaminating reads constitute a fairly low proportion compared to true positive reads, enabling correct calls with high confidence. Taken together, this data suggests that information collected following a 48 h sequencing run is more sensitive, but generally well matched with information collected from the first 10 min.

**Table 6:** Raw read counts (and percent read counts) for false positive assays

Organism	Strain	PCR Assay Number						
		23	28	29	30	07	11	50
*F. tularensis*	239			8917 (0.2842%)	23,584 (0.7516%)			
*F. tularensis*	240				30,616 (1.0196%)	24 (0.0008%)		
*F. tularensis*	241			11,037 (0.3579%)		59 (0.0019%)		
*Y. pestis*	113			22 (0.0009%)				
*Y. pestis*	114	25 (0.0025%)	51 (0.0025%)	3 (0.0001%)			3417 (0.1655%)	
*B. mallei*	164							4 (0.0093%)

Sample read counts combined across concentrations and conditions to give total FP count and percentage per assay

### Comparison of sequence data to real-time PCR

The real-time PCR false negative results for *Francisella* 240 (assay 29) and *Yersinia* 114 (assay 14) (red boxes in Fig. 1) turned out to be true positives in the sequence data at all concentrations. As mentioned above, this suggests that there may have been an issue with detection of the NED fluorophore during the PCR runs for these samples. Curiously, there are no reads in the sequencing data to corroborate the one false positive result seen in the real-time PCR for *Burkholderia* 197 (assay 49), highlighting the importance of including multiple targets for the same strain in the decision-making process.

While highly sensitive sequencing data can correct false negative PCR results, it also appears to cause increased rates of false positives as described above (Table 6). *Francisella* strains 239 (assays 29 and 30), 240 (assay 30), and 241 (assay 29), for example, have mapped reads in assays specific for other *Francisella* strains, especially at lower spike concentrations (Fig. 3). Since these specific false positive amplicon sequences are not found in the whole genome reference sequences (de novo assemblies) of the spiked organisms (Table 7), it is assumed they are due to cross contamination during sample preparation or later steps, and not near-neighbor homologies or other alignment-related issues.

In addition to providing higher resolution of target amplicons, sequencing data also allows for the estimation of target copy number. Differences in read counts between chromosomal and plasmid targets are prominent, as shown in Table 8. Assay 14 (plasmid target) and 15 (chromosomal target) for *Yersinia* 114, for example, have 76 and 9% read abundances, respectively (calculated for each strain by dividing total QC reads mapping to a particular assay by total QC reads mapping to all assays). Assays 01 and 04 (plasmid targets), and 07 (chromosomal target) for *Bacillus* 708-*gi* have read abundances of 42, 55, and 3%. These significantly higher read abundances are indicative of a target on a plasmid in high copy number compared to chromosome.

### Multiplex real-time PCR and sequencing of isolate agents and mixed primer/probe cocktails

**Fig. 4:**
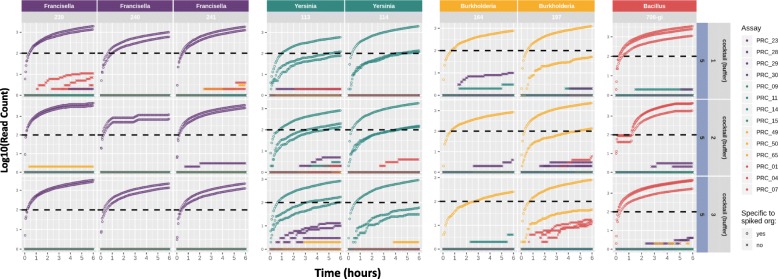
Graphical representation of read counts resulting from multiplex PCR reaction spiked with individual organisms. Read counts per assay per isolate sample during the first 6 h of sequencing are presented in log10 scale. Circles represent expected amplicon reference mapping (TP), crosses represent unexpected amplicon reference mapping (FP). Black dashed line represents an example read count threshold of 100 for True positive call, applied in Fig. 5b for demonstration purposes only

**Fig. 5:**
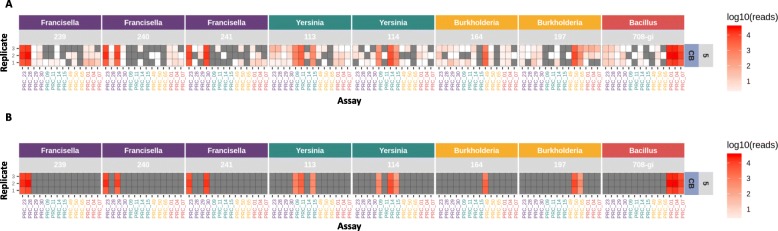
Heat map of sequence read counts from 14 plex real time PCR reactions (48 h data). Heat map of read counts resulting from sequencing of 14-plex PCR assay from single isolate spiked samples on ONT platform (48 h of sequencing data). **a**) Amplicon reference aligned read counts per assay per sample (no read count threshold applied for true positive call). **b**) Read count threshold of 100 applied (all read counts < 100 are reduced to zero, i.e. not called as a positive)

Having determined the baseline performance of limited multiplex PCR (3 to 4 assays in one reaction), we next tested a 14-plex assay. We created a mix of all 14 primer pairs and probes and spiked strains individually at 2.5 X 10^5^ CFU/ml in the respective matrices, extracted DNA and assessed assay performance. Real-time PCR results showed that each spiked strain gave expected results for the corresponding species/strain specific assays (Table 9). The amplicons produced from these 14-plex assays were then sequenced. Figure 4 shows the read count (log_10_ scale) over time, up to six hours of sequencing. For all *Francisella* strains and all but one replicate of *Bacillus* strain, positive detection (≥100 reads) occurs within the first hour of sequencing. Both *Yersinia* and *Burkholderia* strains barely met the 100 read count cut-off for all strain-specific assays within this 6 h time frame, though the higher copy-number target assays surpass this threshold within the first 2 h of sequencing in a majority of replicates. If a read count cut-off for making positive calls is set to ≥1 read (see last subsection of Results), there is a broad range of false positive assay detection. However, this may also be due to barcode cross-talk during de-multiplexing, as the read counts of these false positives after 48 h of sequencing range from only 1 to 75, with a median of 2. This false positive burden could be mitigated by stricter de-multiplexing algorithm parameters. The read count range of true positives is 146 to 31,656 with a median of 4571. Figure 5a and b demonstrate how a read count cut-off of 100 reduces the false positive rate to zero in the 48 h sequence data. It is recognized that this cut-off will need to be adjusted according to the extent of multiplexing and throughput of the selected sequencing platform.

### Multiplex real-time PCR and sequencing of mixed agents and mixed primer/probe cocktails (preparation for set 2)

We next tested if the 14-plex primer/probe cocktail supported the PCR of a mixed agent cocktail. In this case, we combined all eight strains at their highest respective concentration (see Table 2), extracted DNA, and performed 14-plex end-point PCR. Only 4 dyes could be used for any individual real-time PCR run, and the C_*t*_ values from each fluorescent channel represented multiple assays (for example, assays 01, 09, 23, and 49 all used the FAM fluorophore). Although overlapping probes prevent determination of individual assay performance, average C_*t*_ values for FAM, VIC, and NED labeled assays are a few cycles lower (i.e. earlier crossing of the C_*t*_ threshold), respectively, in the mixed agent cocktail as compared to average individual strain cocktail analysis when spiked at the same concentration (Table 10). Coupled with the sequencing data derived from these amplicons which show that all target products are present after end-point PCR, this cumulative effect in terms of C_*t*_ value when the same fluorescent channel is used for multiple assays indicates that they may be performed concurrently without significant reduction in individual assay efficiency.

Interestingly, the CY5 labeled assays did not show a similar corresponding reduction in average C_*t*_ value; instead, the mixed assays have a negative effect compared to individual assay performance. Additional experiments are required, but this discrepancy is likely an artifact of real-time PCR analysis. Only two of the fourteen assays employ CY5 and when combined, all fourteen assays compete for limited reagents (i.e., Taq, Mg++, dNTPs, etc.). This competition, in conjunction with strain specific spiking levels could artificially reduce CY5 detection. Further optimization of PCR conditions would likely resolve this issue.

### Sequencing of set 2 amplicons

**Fig. 6:**
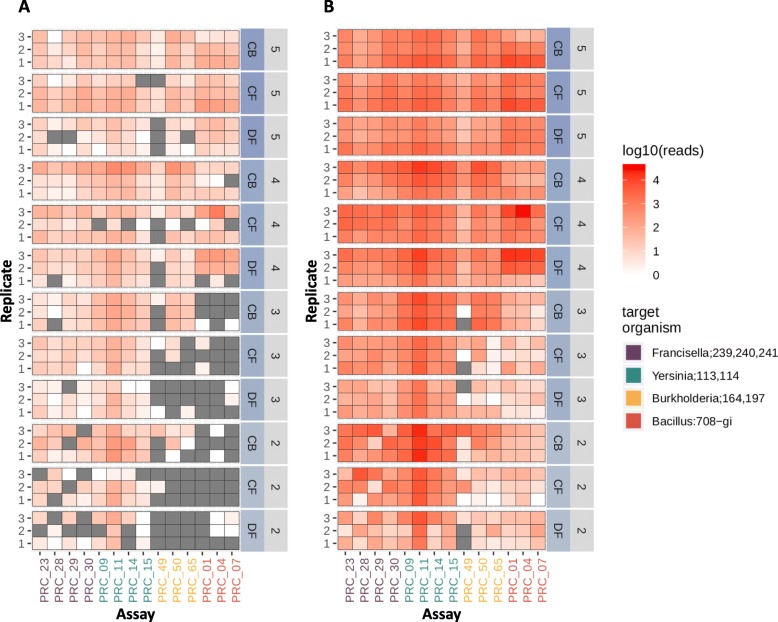
Heat map of sequence read counts from 14 plex real time PCR reactions from samples spiked with all strains (48 h data). Heat map of read counts (log10 scale) of Set 2, mixed agent multiplex amplicon sequencing on ONT platform. **a**) Results analyzed after the first 10 min of ONT data collected and **b**) Results analyzed after the full 48 h runtime. Organism and corresponding strains are indicated across the top, condition and spiking concentrations (CFUs) (10 fold dilution steps 5 through 1) are along the right side (numbers in Table 2), and replicate number along the left side. Conditions are as follows: CB cocktail buffer, CF clean filter, and DF dirty filter. The x-axis indicates assay (or amplicon reference)

To determine whether ONT sequencing of the amplicons produced using the 14-plex PCR assay would provide higher sensitivity information than PCR alone, we utilized the same cocktail of all 8 strains, and tested different spiking concentrations, yielding 36 unique samples (4 concentrations × 3 matrices × 3 replicates). These mixed sample DNA extracts were split into 2 groups (known and blinded for the sequencing team), and both were amplified using all 14 sets of primers and probes in a single reaction. The resulting amplicons were barcoded and run on 2 flow cells. Since the groups were identical, the sequence data from the two groups were combined for analysis. Data from the first 10 min and the full 48 h of sequencing are shown in Fig. 6a and b, respectively. There was a similar pattern of read counts and true positive detection as spike concentration decreases in these mixed amplicon samples as was observed for individually spiked samples. Read data collected after 10 min of sequencing shows LoDs for *Francisella* and *Yersinia* targets of ~ 100–250 CFU/ml and moderately higher LoDs for *Burkholderia* and *Bacillus* targets of ~ 2500–5000 CFU/ml. All targets were detected in the mixed samples, but there were inherent differences in performance (total assay read count per sample) in the multiplex reaction that is also seen in singleplex reactions (Additional file 2: Table S2). After 48 h of sequence data collection, every assay target was detected in all conditions (matrix background, spike concentration) with the exception of assay 49 (targets *Burkholderia* 164) which also exhibited a PCR efficiency > 100% (148.11%) (Table 4), indicating less optimal amplification. An ideal PCR reaction would exhibit an efficiency of 90–110% efficiency reflecting an increase in PCR product by two-fold every cycle. This data suggests that the 14-plex assay works well and exhibits similar limits of detection as the singleplex (Additional file 2: Table S2) or limited multiplex single agent end-point PCR (Table 4).

As a control, unamplified mixed agent cocktail extracts were also prepared and sequenced to determine if the assay targets could be detected among resulting reads without a PCR step (Table 1, Set 2, all agents (unamplified)). Only two of these samples contained reads that mapped to one of the reference sequences, and only a single read in each sample mapped in each case. Surprisingly, both were found in a clean filter matrix (it was assumed if detection occurred it would likely be in the cocktail buffer matrix due to higher extraction efficiency). One read was identified in a sample spiked at the lowest concentration (assay 11 for *Yersinia* 113) and the other was identified in a sample spiked at the highest concentration (assay 01 for *Bacillus* 708). This data suggests that even at spike levels of ~ 10^5^ CFU, an initial PCR amplification is still necessary for reliable positive detection of target amplicons specific for any given strain.

### Analysis of matrix background

As some of the spiked agents were applied to filters with “background” genetic material (dirty filter samples), analysis of the non-target sequences was also performed. The results for the 48 h data from all samples of Set 1 and Set 2 are presented in Additional file 2: Figure S2 and S3, respectively. Sequencing reads were combined across the 5 concentrations and grouped by organism and condition, resulting in 27 unique samples in set 1, and 9 unique samples in set 2. These reads were binned into four categories: amplicon_mapped reads that mapped to an assay amplicon reference; contig_mapped, reads that did not map to any assay amplicon reference that then mapped to a set of reference whole genome sequence of all spiked organisms (the whole genome sequences of the spiked organisms were generated using Illumina platform); classified, reads that did not map to any of the assay amplicon references or reference genomes that then classified via Kraken (v1) [17] using a RefSeq database; and unclassified, reads that remained unclassified via Kraken. Combined QC read counts ranged from 249,092 to 9.6 million, with a median of 1.2 million. While total read counts across all conditions varied, there is a general, and unsurprising, trend toward higher counts in samples recovered from dirty filters.

It was anticipated that sequencing-based detection of specific sequences would be more difficult in samples extracted from a complex/ “dirty” matrix background with more background reads than clean filter or cocktail buffer samples. Additional file 2: Figure S2 supports the assumption that the proportion of reads mapped to an assay target (i.e., “amplicon_mapped” reads) increases as condition complexity decreases in a majority of cases (CB > CF > DF). The amplicon mapped reads varied from near 0 to 93% of total sample reads. The average basecall quality (phred33) and read length for all “contig_mapped” reads are 12.6 and 2726 bp (base pairs), respectively. In all samples, the majority of remaining reads (those reads not mapping to amplicon or contig references) remain “unclassified”, and the average basecall quality and read length for this post-mapping read group is only 8.1 and 92 bp, respectively. This significant reduction in both basecall quality and read length is likely due to the chemical or irradiation inactivation step for all spiked organisms except *Bacillus (708-live)*, and may explain why these reads could not be mapped to a contig reference or classified using Kraken.

### Determination of the sensitivity, specificity, and precision of real-time PCR and sequencing approaches

**Fig. 7:**
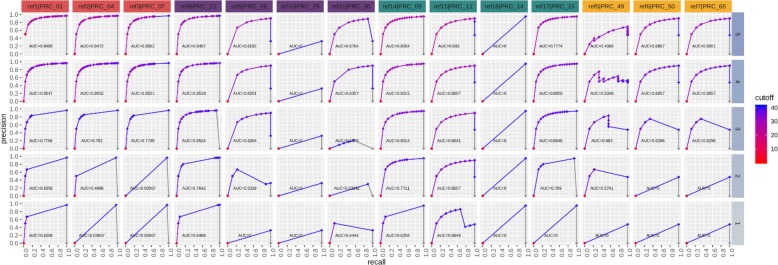
Real-time PCR precision-recall (PR) curves. PR curves are shown for each of the 14 assays tested (faceted by column), split over the 5 steps of the dilution series (faceted by rows). Replicates are kept separated for this analysis. The C_*t*_ value cutoff for calling positive/negative is ranged from 0 to 40 in steps of 0.1. The area under the curve (AUC) is calculated using the ‘step’ method. Most assays achieve near 100% precision and 100% recall at the standard Ct value cutoff of 40 for the highest 3 concentrations (3,4, and 5), with the exception of PRC_29, PRC_30, and PRC_14

**Fig. 8:**
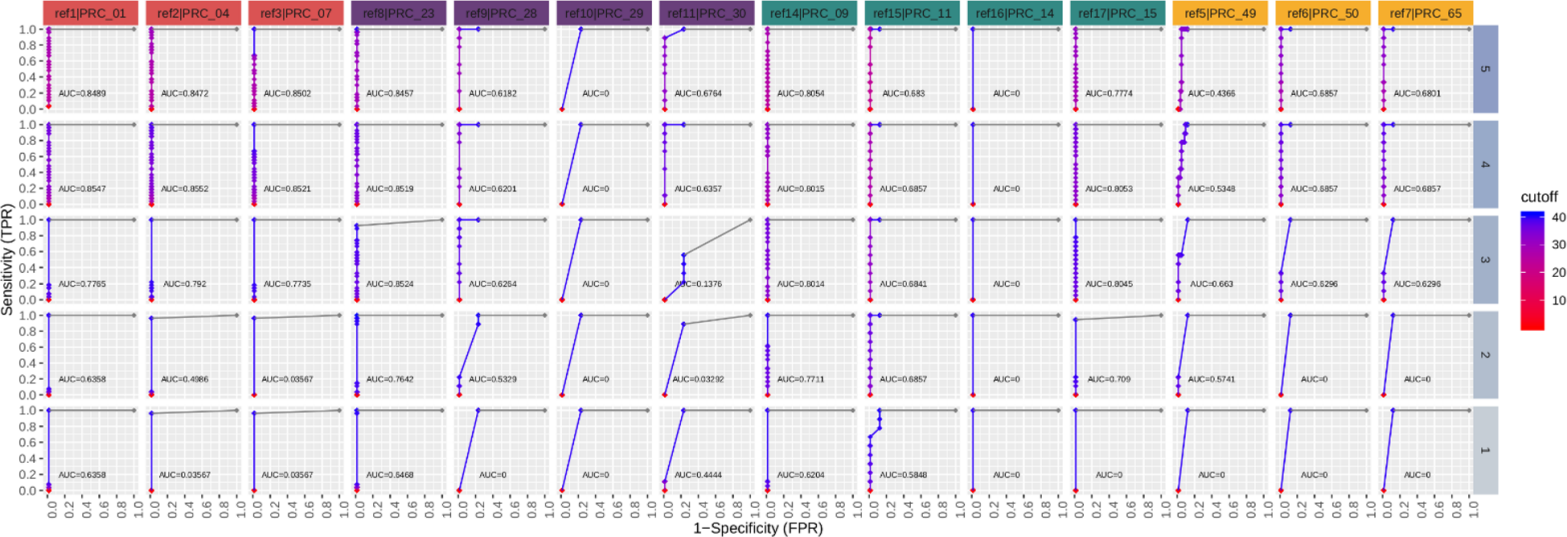
Real-time PCR receiver operating characteristic (ROC) curves. Curves are shown for each of the 14 assays tested (faceted by column), split over the 5 steps of the dilution series (faceted by rows). Replicates are kept separated for this analysis. The Ct value cutoff for calling positive/negative is ranged from 0 to 40 in steps of 0.1. The area under the curve (AUC) is calculated using the ‘step’ method. Most assays achieve a 100% sensitivity and 0% FPR at the standard Ct value cutoff of 40. The FPR for all assays remains at or very near zero across all cutoffs

**Fig. 9:**
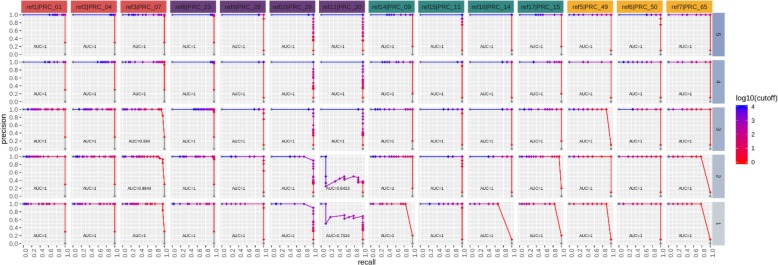
ONT sequencing PR curves. Curves are shown for each of the 14 assays tested (faceted by column), split over the 5 steps of the dilution series (faceted by rows). Replicates are kept separated for this analysis. The read count cutoff for calling positive/negative is log10 scaled. The area under the curve (AUC) is calculated using the ‘step’ method. Most assays achieve 100% precision and 100% recall near a read count cutoff of 100 for the highest 3 concentrations (3,4, and 5), and many achieve these perfect scores at the lowest 2 (1,2) with the exception of PRC_30

**Fig. 10:**
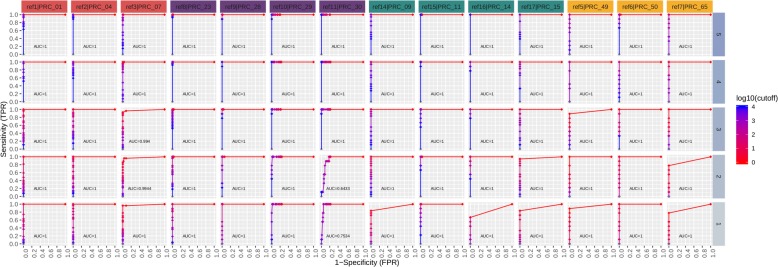
ONT sequencing ROC curves. Curves are shown for each of the 14 assays tested (faceted by column), split over the 5 steps of the dilution series (faceted by rows). Replicates are kept separated for this analysis. The read count cutoff for calling positive/negative is log10 scaled. The area under the curve (AUC) is calculated using the ‘step’ method. Most assays achieve a 100% sensitivity and 0% FPR near a read count cutoff of 10 for the highest 3 concentrations (3, 4, and 5), and many achieve this AUC at the lowest 2 (1, 2), with the exception of PRC_30

The standard cut-off threshold for calling a real-time PCR positive is C_*t*_ value of < 40. To determine the sequencing read count thresholds required to make a high confidence true positive call, we used Precision-Recall (PR) and Receiver Operating Characteristic (ROC) curves. This analysis was performed on sequence data grouped by matrix type and spike concentration, and then interrogated per assay. Definitions for metrics used for real-time PCR and sequencing calls are shown in Table 11. Figures 7, 8, 9, 10 depict PR and ROC curves for each of the 14 assays tested (identified at top and colored for organism), split over the 5 steps of the dilution series (right side, faceted by rows, decreasing from top to bottom). Replicates of samples were kept separated during the thresholding and data generation process. The read count cut-off for calling a positive/negative result for the sequence data is represented in log^10^ scale. The area under the curve (AUC) is calculated using the step method in the AUC function of the R package DescTools (v0.99.19) [18]. As a bench mark, the standard cut-off threshold for calling a real-time PCR positive is C_*t*_ < 40. Most assays with only a single spiked agent achieve near 100% precision and 100% recall at this traditional C_*t*_ value cutoff, for the highest 3 concentrations (Set 1, Steps 3, 4, and 5, Table 2), with the exception of assays 29, 30, and 14. Likewise, most assays maintain a 0% (or very close to 0%) false positive rate (FPR) across all C_*t*_ cut-offs. For sequencing, there is no specific cut-off value established for a positive call, but based on the results presented here for the 48 h sequencing data, a read count cut-off of 100 achieves a 100% precision and 100% recall for most assays at the same 3 highest concentrations, and many achieve these perfect scores at the lowest 2 concentrations with the exception of assay 30.

**Fig. 11:**
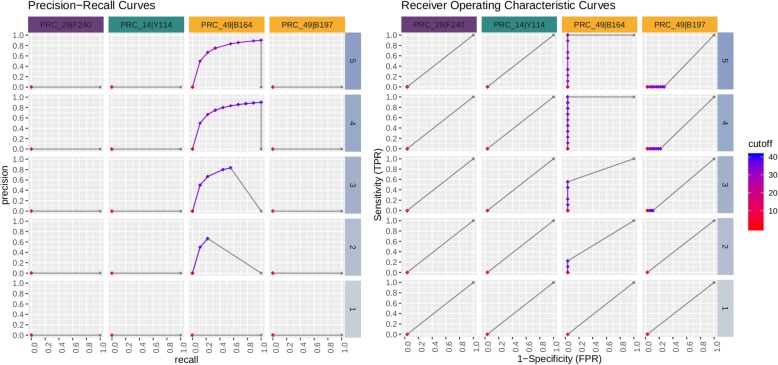
Real-time PCR PR and ROC curves for assays having a real time PCR false negative or false positive. Real time PCR did not detect either PRC 29 or 14 for F240 and Y114, respectively, resulting in a 0% precision and recall for all tested cutoffs. PRC 49 was detected in B164 and was expected to, and at the 2 highest concentrations achieves 100% recall and close to perfect precision. However this assay was not expect to be detected for B197, resulting in non-zero FDR for the top 3 highest concentrations. Refer to Table 4 for additional interpretation

**Fig. 12:**
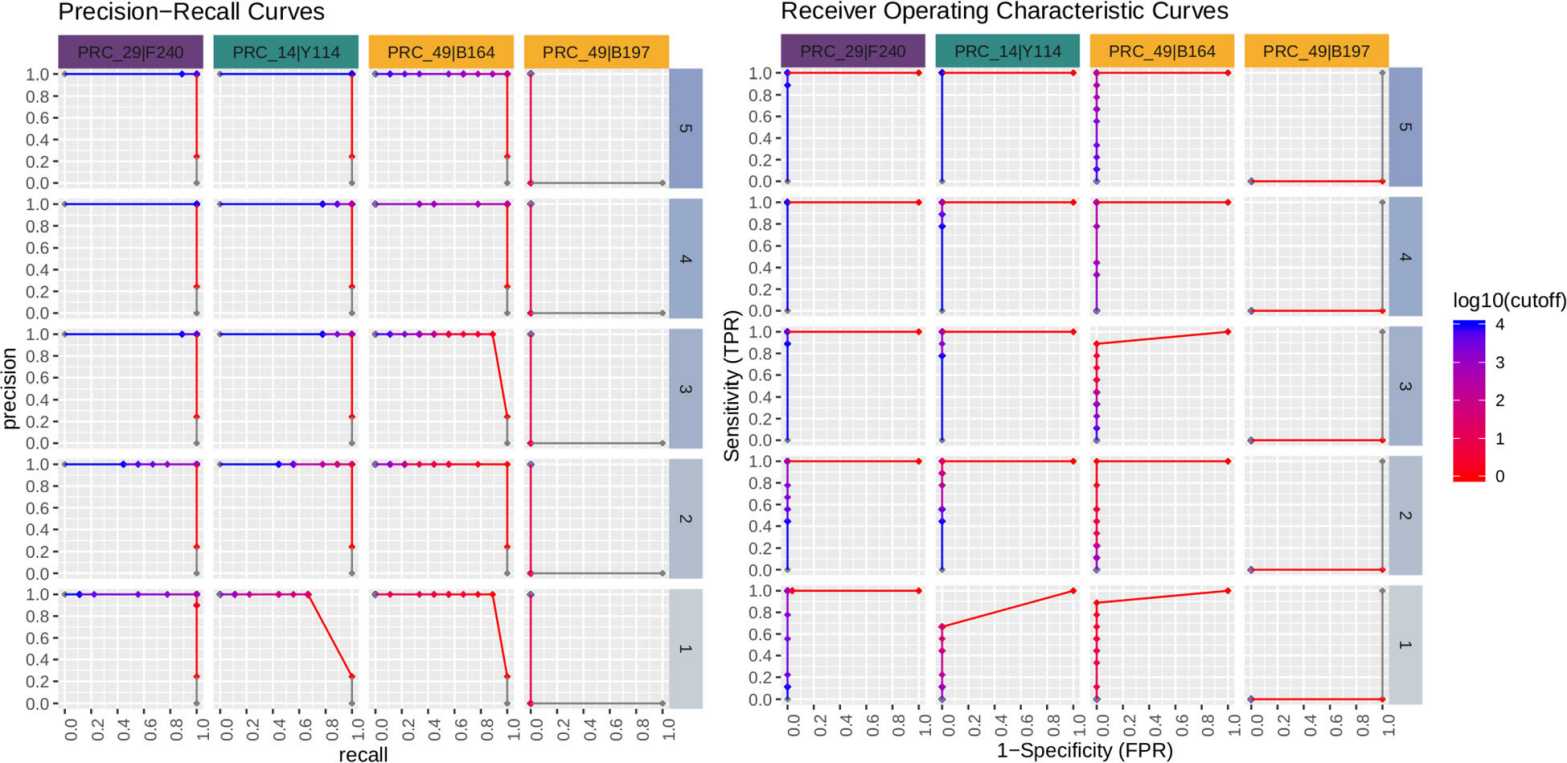
ONT sequencing PR and ROC curves for assays having a real time PCR FN or FP. Metrics for these particular assays are not significantly changed when separating out organisms per assay and in most cases, analysis of sequenced amplicons produced by these assays results in perfect precision, recall, and FDR. Refer to Tables 4 and 11 for additional interpretation

The data for the 3 specific assays which produced a false negative or false positive result in real-time PCR were separated and used to generate a PR/ROC curve per respective assay-organism pairing (Figs. 11 and 12). Assay 29 and 14 for detection of *Francisella* 240 and *Yersinia* 114, respectively, were not detected in real-time PCR (false negatives), and therefore the precision and recall remain at 0% for all C_*t*_ cut-off values at all spike concentrations. However, these values quickly reach 100% in the sequence data at read cut off values in the range of 10 to 100 reads, down to the second lowest spike concentration. Assay 49 is expected to be positive for *Burkholderia* 164, which for this specific pairing is borne out in both the real-time PCR and sequencing data. An interesting pattern is revealed in the real-time PCR results as the spike concentration is reduced, the precision drops to 0% at lower C*t* cut-off values and is undetected at the lowest concentration. Again, the precision and recall statistics from sequencing data for this assay reach high values at the same read count cutoff range. Lastly, when analyzing data from *Burkholderia* 197 for assay 49, real-time PCR has non-zero FPRs at the 3 highest spike concentrations, and 0% precision and recall since the assay is not expected not detect this organism. Contrary to PCR results, amplicon sequence from assay 49 are not present in the sequencing data, and the PR and ROC curves remain flat across the entire read count cut-off range examined. In this particular instance, sequencing serves to verify an unexpected real-time PCR false positives result.

## Discussion

### Comparison of real-time PCR and amplicon sequencing

In this study, we have established a baseline for comparing traditional singleplex PCR assays to an amplicon sequencing assay using the ONT MinION device. It is clear from the data presented in this study that even within the first 10 min of a MinION sequencing run, an operationally relevant “positive detection” call can be made at a high confidence even if the target organism was present at a low concentration. The only exception to this finding was the *Burkholderia* strains, perhaps due to differences in PCR efficiencies between assays. Given the full 48 h of sequencing time, the MinION data analysis returns true positive calls for nearly all assays at all concentrations for all organism targets.

Here, we only compared the performance of currently used singleplex real time PCR assays, limited multiplex (3 or 4 plex) real time PCRs and a 14 plex end point PCR to Nanopore amplicon sequencing. The focus of the study was to explore the feasibility of deploying Nanpore sequencing as a point of care/point of need device. There are other multiplex PCR platforms such as digital PCR (dPCR) with even more multiplex and high throughput capabilities. Comparison of dPCR performance, in future studies, to Nanopore amplicon sequencing, would be extremely valuable. However, the use of dPCR as a field deployable platform has not been demonstrated yet and thus limits its utility to a fixed lab rather than a point of care /point of need application.

Despite these promising findings, there are some limitations to the amplicon sequencing approach that need to be addressed. First, the pipeline described here is still time-consuming; i.e., > 12 h total for the preparation of 45 samples, PCR, sequencing, and analysis. This process can be improved by simple protocol modifications such as adding the barcode and sequencing primer sequences in the target specific primer sequences (tailed PCR) to reduce the time from sample to sequence information. Additionally, the sequencing assay can sometimes prove “too sensitive” in the sense that false positive reads are observed in the sequencing data (0.0001 to 1.0196% of total reads in samples not expected to contain those reads). This may be indicative of cross contamination of even a small aerosolized droplet during sample preparation especially when processing multiple samples (up to 45 samples in some cases) and is further exacerbated by the second PCR step required for barcoding of the samples during library preparation. We believe that this may not be an issue in scenarios where real field samples are not expected to contain multiple agents in the same sample. Establishing better procedures to minimize aerosols during sample preparation and removing the second amplification step by directly ligating barcodes onto amplicon ends or having tailed primers during PCR could remedy this issue while simultaneously reducing the total sample-to-answer time closer to 8 h. Additionally, there were some false negative PCR results at low spike levels. Differences in the assay efficiencies could have contributed to PCR false negatives. Currently, PCR efficiency of assays in individual vs multiplex format is not fully understood. We posit, that assays 14 and 29 are not as robust as their agent counterparts (assays 09, 11, and 15, and assays 23, 28, and 30, respectively). However, combination of all primer/probe sets could sufficiently alter reaction dynamics to allow for some assays to effectively compete for limited PCR reagents and suppress others such as 14 and 29. Clearly, further investigation and optimization are required for multiplex assay optimization and preventing cross contamination.

Current error rates (at the time of experiment) of the R9 ONT flowcell is approximately 5–10%. In other words, approximately one in every ten base calls is expected to be incorrect. Since our amplicons are ~ 100 bps, that leaves around 90 bps for alignment to the limited database of reference amplicon sequences, which does not pose a problem for miss-identification if used in conjunction with an appropriate mapping quality filter. In this study, a MAPQ filter of 60 was applied to all alignments, which is the absolute highest MAPQ reported for an alignment made using the BWA algorithm. Additionally, with our high sequencing depth, we could identify SNPs within a detected amplicon.

### Analysis of target amplicon sequences

There are some advantages to the amplicon sequencing approach for pathogen detection that are not provided by traditional real time PCR assays; for example, detection of engineered threats and natural variants, plus very high multiplex and high-throughput. In addition to simple detection of specific amplicons via read mapping to amplicon sequences, an analysis of variation within each amplicon region can be performed to assess natural variants or engineered threats. In our work, the amplicon sequences (the same set used in the BWA reference database) were mapped to sets of whole genome sequences of the spiked organisms. Detailed results of amplicon sequence alignment are presented in Table 7.

**Table 7:** Mapping of the amplicon sequences to associated genome references

Organism	Strain	PCR Assay	Amplicon Length (bps)	CIGAR*	Insertions	Deletions	SC left	SC right	Alignment Expected?	Expected PCR result	Observed PCR Result
*B. anthracis*	708	PRC_01	110	70M3D40M	0	3	0	0	Y	+	+
*B. anthracis*	708	PRC_04	182	155M4D27M	0	4	0	0	Y	+	+
*B. anthracis*	708	PRC_07	96	96 M	0	0	0	0	Y	+	+
*Y. pestis*	113	PRC_09	68	68 M	0	0	0	0	Y	+	+
*Y. pestis*	114	PRC_09	68	68 M	0	0	0	0	Y	+	+
*Y. pestis*	113	PRC_11	79	79 M	0	0	0	0	Y	+	+
*Y. pestis*	114	PRC_14	103	103 M	0	0	0	0	Y	+	–
*Y. pestis*	113	PRC_15	67	67 M	0	0	0	0	Y	+	+
*Y. pestis*	114	PRC_15	67	67 M	0	0	0	0	Y	+	+
*F. tularensis*	239	PRC_23	135	135 M	0	0	0	0	Y	+	+
*F. tularensis*	240	PRC_23	135	135 M	0	0	0	0	Y	+	+
*F. tularensis*	241	PRC_23	135	133M2S	0	0	0	2	Y	+	+
*F. tularensis*	239	PRC_28	171	90M1D81M	0	1	0	0	Y	+	+
*F. tularensis*	239	PRC_29	119	48S71M	0	0	48	0	N	–	–
*F. tularensis*	240	PRC_29	119	119 M	0	0	0	0	Y	+	–
*F. tularensis*	241	PRC_29	119	71M48S	0	0	0	48	N	–	–
*F. tularensis*	241	PRC_30	126	126 M	0	0	0	0	Y	+	+
*B. mallei*	164	PRC_49	100	100 M	0	0	0	0	Y	+	+
*B. pseudomallei*	197	PRC_49	100	no alignment**						–	+
*B. pseudomallei*	197	PRC_50	115	115 M	0	0	0	0	Y	+	+
*B. pseudomallei*	197	PRC_65	67	67 M	0	0	0	0	Y	+	+

Mapping of the amplicon sequences to the whole genome de novo sequence reference of the spiked strains to detect possible mismatches. All amplicon alignments match expected contig reference, however there are alignments with heavy soft clipping (SC). *Concise Idiosyncratic Gap Alignment Report; S - soft clipping, M - match, D - deletion, I - insertion. Soft-clipped parts of query sequence are ignored when calculating alignment mapping quality (consequence of local alignment). **Shown for completeness and comparison purposes

To highlight the power of sequence variation analysis, *Bacillus* strain 708 contained a 3 and 4 base pair deletion in the regions targeted by assays 01 and 04, respectively. Similarly, if the targets have been engineered on plasmids the read abundance may indicate such anomalies. Based upon assay 01 and 04 relative read abundances when compared to assay 07 (genomic target), these mutant assay targets are likely on a high-copy plasmid pXO1 and pXO2 (Table 8). Since the deletion occurred in the center of the amplicon they did not affect the efficiency of the PCR or amplicon sequencing. However, sequencing provided additional information that would not have been obtained in the PCR itself. Also, in performing environmental surveillance based on PCR, one might encounter a number false positive detection events due to near neighbor hits. Subsequent sequencing of the amplicons can shed light on the nature of these hits providing in depth sequence information. These hits may have perfect matches to the PCR signatures (primers and probes) but have mismatches in the rest of the amplicon sequences.

**Table 8:** Mapped read abundances per assay amplicon for each spiked organism

Organism	Strain	PCR Assay Number													
		23	28	29	30	09	11	14	15	49	50	65	01	04	07
*F. tularensis*	239	0.32	0.68												
*F. tularensis*	240	0.52		0.48											
*F. tularensis*	241	0.48			0.52										
*Y. pestis*	113					0.21	0.61		0.18						
*Y. pestis*	114					0.15		0.76	0.09						
*B. mallei*	164														
*B. pseudomallei*	197										0.90	0.10			
*B. anthracis*	708-gi												0.42	0.55	0.03
*B. anthracis*	708-live												0.43	0.53	0.04

Mapped read abundances (range 0 to 1) per assay amplicon for each spiked organism. Reads summed across conditions, concentrations, and replicates

**Table 9:** Real-time PCR results of 14-plex assay

Organism	Strain	Agent	*F. tularensis* assays				*Y. pestis* assays				*Burkholderia* assays			*B. anthracis* assays		
*F. tularensis*	239	A	30.63	32.97	TN	TN	–	–	–	–	–	–	–	–	–	–
	240	B	26.05	TN	28.75	TN	–	–	–	–	–	–	–	–	–	–
	241	C	29.80	TN	TN	30.97	–	–	–	–	–	–	–	–	–	–
*Y. pestis*	113	D	–	–	–	–	24.86	23.04	TN	30.28	–	–	–	–	–	–
	114	E	–	–	–	–	27.75	TN	29.71	31.04	–	–	–	–	–	–
*B. mallei*	164	F	–	–	–	–	–	–	–	–	32.81	TN	TN	–	–	–
*B. pseudomallei*	197	G	–	–	–	–	–	–	–	–	TN	32.60	32.14	–	–	–
*B. anthracis*	708	H	–	–	–	–	–	–	–	–	–	–	–	27.07	28.45	28.97
PCR Assay Number			**23**	**28**	**29**	**30**	**9**	**11**	**14**	**15**	**49**	**50**	**65**	**01**	**04**	**07**
Probe Dye Channel			**FAM**	**VIC**	**NED**	**CY5**	**FAM**	**VIC**	**NED**	**CY5**	**FAM**	**VIC**	**NED**	**FAM**	**VIC**	**NED**

Real-time PCR results using a mixed assay of all 14 sets of primers and probes tested on individual agents. Each sample contained a single agent, extracted in singlet and analyzed by PCR. Agent concentration are all at 2.5E+ 05 CFU/mL. Each PCR reaction employed all 14 primer/probe sets. Data shows that only agent specific primer/probe sets detected with no FP or FN. Values in the cells indicate the C_t_ value of an observed positive result (C_t_ < 40) where a positive result was expected. A minus sign (−) indicates the assay-organism combination was not tested. Cells containing TN indicate an observed negative or undetected result (C_t_ ≥ 40) where a positive result was *not* expected

**Table 10:** Real-time PCR results of mixed agents and assays

Probe Dye Channel	Extraction 1	Extraction 2	Extraction 3	Average Ct Set 2	Average Ct Set 1*	Difference**
FAM	22.56	24.00	24.46	23.67	28.02	3.56
VIC	22.36	23.32	23.55	23.07	28.02	4.47
NED	25.36	26.53	26.98	26.29	28.96	2.67
CY5	30.85	33.28	29.69	31.27	29.82	−1.45

Real-time PCR results of mixed agent samples using primer/probe cocktails. All 8 organism strains are included in the mixutre. *Averages of C_t_ values among each probe dye channel from Table 9. **Difference between mixed and individual probe results (average *Set 1* C_t_ values minus average *Set 2* C_t_ values)

**Table 11:** Definitions used for metrics calculations

Metric	Description	Function
True Positive (TP)	Result is positive, key is positive	
True Negative (TN)	Result is negative, key is negative	
False Positive (FP)	Result is positive, key is negative	
False Negative (FN)	Result is negative, key is positive	
Precision (PPV)	Positive Predictive Value; number of TP of result positives	TP/(TP + FP)
Recall (TPR)	Sensitivity; number of TP out of all key positives	TP/(TP + FN)
Specificity (TNR)	Number of TN out of all key negatives	TN/(FP + TN)
False Positive Rate (FPR)	Number of false positives out of all key negatives	FP/(FP + TN)

Each sample from real-time PCR and sequencing was measured for a target assay (columns faceted along top of Figs. 7, 8, 9, 10). Under Description, ‘Result’ is in reference to a real-time PCR C_t_ value or a sequence mapped count above (negative for PCR, positive for sequencing) or below (positive for PCR, negative for sequencing) a particular threshold. These definitions were used to generate the Precision-recall (PR) and Receiver Operator Characteristic (ROC) curves in Figs. 7, 8, 9, 10. A range of thresholds was employed for the generation of PR and ROC curves

In our analyses we counted only reads that were significant matches to a reference sequence (MAPQ> = 60). Reducing this threshold may pull out spurious hits that would reveal false positives of interest. High multiplex PCR also allows for including redundant assays in the same reaction to make high confidence sequence-based calls on the targeted organism vs near neighbors. For example, including a *Francisella* screening assay along sub speciation tests (*tularensis*, *holartica* and *novicida*) in one sequencing assay would resolve such false positive hits without having to perform additional PCR assays. If properly designed, one can expand the panel of the amplicon sequencing assay by including not only detection assay but also other markers such as pathogen specific antibiotic resistance genes. Also, barcoding allows for processing multiple samples in the same sequencing run.

## Conclusions

Overall, in this study we have established that 1) biodetection via amplicon sequencing is more sensitive than real-time PCR alone, especially when the target agent is present at low target concentrations in the sample, 2) detection via amplicon sequencing in *multiplex* experiments (Fig. 4) is as successful as in *singleplex* experiments (Figs. 2 and 3) and 3) multiplexing even at this relatively small scale is not possible with real-time PCR due to limitations in fluorophores.

The ONT MinION based amplicon sequencing assay is so sensitive that it amplifies some of the problems due to cross contamination of samples that one would not see in the conventional real time PCR assays.

We established a pipeline for one specific application: environmental sample collection filter, nucleic acid extraction from filter, PCR, and sequencing. The routine takes more than 12 h for processing up to 45 samples with the current protocols. The goal of our future studies will be to reduce the entire process to less than 8 h from sample processing to sequence analyses and making an actionable call.

One of the key bottlenecks in metagenome-based sequencing for pathogen detection in samples is the vast enormity of the data produced and the bioinformatics infrastructure and expertise needed to process the data and linking the causative agent to disease. The amplicon sequencing approach does not have such requirements since the goal is to detect a pre-selected panel of agents.

Our future studies will focus on improving and optimizing the sample preparation including DNA extraction and sequencing protocols to reduce the time frame and preventing false positive and false negative instances.

## Methods

### Overview

**Fig. 13:**
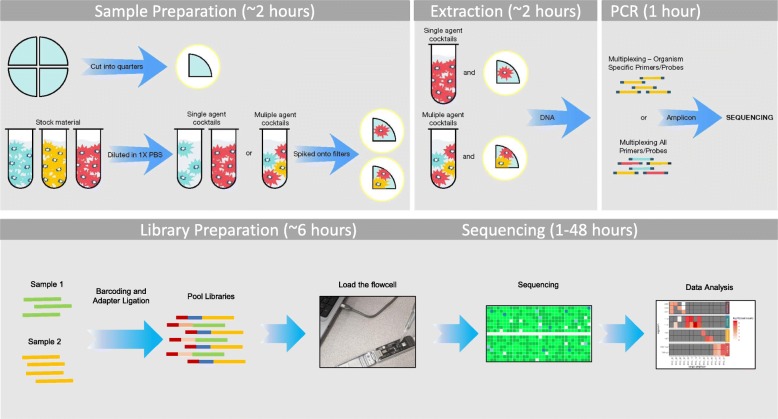
Illustration of sample to sequence workflow and estimated times for each step. Sample preparation, extraction, PCR, library preparation, and sequencing work flows withestimated times are shown. Both the PCR workflow (top) and sequencing workflow (bottom) include preparing and extracting samples in batches of 45 (9x) and 32 (3x) samples in parallel. In this study, a total of 12 ONT flow cells were used to sequence 513 samples. See Table 1 for a detailed breakdown of sample sets. See Table 2 for a list of spike concentrations. Clip art, photo and screenshots depicted are from the authors

Sample preparation, PCR, and sequencing work flows are illustrated in Fig. 13. A list of pathogens and corresponding assay details are presented in Table 3. In total, 513 samples were prepared and run on 12 ONT flow cells. The set 1 experiment consisted of 405 samples (9 plates of 45 samples). Each plate tested a single agent spiked at 5 different concentrations on to 3 different matrices (CB, CF, and DF) in biological triplicate (5x3x3 = 45). The set 2 experiment consisted of 108 samples (3 plates of 36 samples). Each set 2 plate tested 8 combined agents spiked at 4 different concentrations on to the same 3 matrices, again prepared in triplicate (4x3x3 = 36). The first two set 2 plates contained amplified DNA of known or blinded composition, and the third plate contained known, but unamplified DNA extracts. Table 1 presents a breakdown of these sample sets. Table 2 lists the concentrations of the various spiked agents.

### Sample preparation and PCR amplification

Cocktail (PBS) and filter samples were prepared in accordance with an established sample preparation protocol. Briefly, serial dilution of individual, inactivated stocks of each agent were added to 1X PBS and nucleic acids were either extracted directly from the PBS (termed “buffer cocktails”) or spiked onto quarter filters (3 μm pore size, 47 mm hydrophobic polytetrafluoroethylene (PTFE) membrane, Millipore catalog# FSLW04700). Filter samples were allowed to dry and then nucleic acids were extracted as “filter extracts” (Fig. 13). Both clean and artificially dirty (contaminated with environmental background) filters were employed.

Cells or spores were lysed via mechanical disruption using a bead beater prior to extraction. Nucleic acids from single agent cocktails and associated spiked filters were extracted in high-throughput format via vacuum filtration and 96-well extraction plates (Millipore catalog# MSGVN2250, followed by MSGVN03050). Nucleic acid from combined agent cocktails and associated spiked filters were extracted using individual centrifugal filter units (Millipore catalog# UFC30GV0S followed by UFC5030BK). All extracts were washed three times with Tris-EDTA prior to elution in molecular-grade water. DNA extracts were heat-inactivated and PCR was performed using the ABI 7500 platform (Thermo Fisher Scientific).

Single agent samples and filter extracts were initially amplified through multiplexing of only organism-specific assays to validate assay detection performance. Subsequently, single agent samples and filter extracts were amplified through multiplexing of all assays (all primers and probes in one PCR reaction) to validate assay specificity and examine cross-reactivity. Multiple agent samples and associated filter extracts were also analyzed through multiplexing of all assays. Amplified products were used for subsequent sequencing analysis.

### Library preparation and sequencing using GridION™

ONT sequencing libraries were generated from PCR amplicon samples and sequenced using the Oxford Nanopore GridION™ instrument. Sample concentrations were not normalized prior to library preparation so sequencing results would reflect experimental conditions and variables. Finished libraries were quality checked using the Agilent 2200 Tape Station System and the Thermo Fisher Qubit 4 Fluorimeter. Equal volumes of finished libraries were pooled together and processed for sequencing. The library preparation illustrated in Fig. 13 covers the QC steps, which included the Agilent 2200 Tape Station System and Thermo Fisher Qubit 4 Fluorimeter. The time listed for library preparation includes these steps.

Sequencing of Set 1 amplicons (single agent samples) was performed by multiplexing up to 45 samples on a single flow cell using the ONT PCR Ligation Kit (SQK-LSK109) with the ONT Barcoding Expansion Kit (EXP-PBC096). A maximum of five flow cells were run simultaneously on the ONT GridION instrument. These same flow cells can alternatively be run one at a time on the ONT MinION. Set 2 amplicons (multi agent samples and unamplified samples) were also sequenced in multiplex fashion, pooling up to 36 samples on a single flow cell. The ONT FLO-MIN106D R9.5 flow cells were used for all sequencing runs. All steps were followed according to the PCR barcoding (96) genomic DNA ONT protocol (SQK-LSK109, version: PBGE96_9068_v109_revG_23May2018).

### Post sequencing analyses

Raw ONT signal data was base-called using the Guppy Base calling Software Version 2.3.7, (available to ONT customers via their community site). Resulting FASTQ files were then de-multiplexed by barcode followed by adapter and quality trimming using Porechop [19]. Demultiplexed sample FASTQ files were then aligned to an amplicon reference or genome reference database using the Burrows-Wheeler Aligner (BWA) [16]. The BWA database used to map amplicon sequences is a BWA index of the fasta containing the 14 amplicon sequences (hence a closed database) assayed in this study. Due to the extremely small sequence space of the input fasta file used for indexing, MAPQ of alignments are likely skewed higher than they otherwise would be. A mapping quality cutoff of MAPQ ≥60 was applied (the highest MAPQ reported by BWA) to the resulting bam files using samtools [20], and a read count was tabulated per amplicon reference. Background reads (i.e. non-target amplicon reads) were classified using the metagenomics sequence classification tool Kraken (v1.0) [17]. The Kraken database was built from references of all viral and bacterial sequences from RefSeq. ONT sequences were binned by output time using the custom BASH script nanotimeparse (https://github.com/raplayer/nanotimeparse.git). All figures were generated using ggplot2 [21] in the R Project for Statistical Computing software [22]. The authors note that GNU Parallel was critical to the timeliness of the analyses [23]. GNU parallel is a Linux program that enables parallelization of CPU processes. In this study, custom BASH parsing and aggregating functions were used extensively on resulting BWA alignment files, which would have taken considerably longer had these processes not been run in parallel fashion.

### Precision-recall and receiver operating characteristic curves

Precision-Recall and Receiver Operator Characteristic (ROC) curves were generated using real-time PCR C_*t*_ values and sequence read count per assay in order to better compare true positive signals between assay methods. A standard threshold for making a positive detection call using sequence read data is not currently well-established. We therefore hope this comparison approach will aid in determining an appropriate read cut-off approximately equivalent to the standard C_*t*_ value < 40 used for calling positives in real-time PCR. Preprocessing of data combined results from multiple organisms that were interrogated using the same PCR assay, meaning the total count of positives and negatives will vary per assay. Additionally, for this analysis, it is important to note that the total number of true and false positives and negatives is dependent on the number of cut-off thresholds tested. For both real-time PCR and sequencing data, each data point in each plot represents the x and y (recall and precision, or false discovery rate (FDR) and sensitivity) values of a particular assay at a particular C_*t*_ or sequence read count cut-off threshold. For example, if a spiked organism that should be positive for assay 14 has a C_*t*_ value of 40.1, and the standard positive result cut-off threshold of 40 is applied, a false negative result would be returned. Inversely, for sequencing data, if the read count for assay 14 is 100, and the read count cut-off threshold is 50, a true positive would be counted. This same threshold is applied for every sample in the set, and once all true and false positive and negative counts have been totaled for an assay, the precision, recall, and FDR are calculated. This procedure is then repeated for multiple cut-off thresholds; C_*t*_ value = 0 to 41 in steps of 0.1 for real-time PCR, and read counts = 0 to 10,000 in varying steps for sequencing. It should be noted that a proportional read count threshold is likely more appropriate than a static read count cut-off to account for the time variant nature of ONT sequencing output which lends itself to real-time analysis. This method will be investigated in future studies.

## Supplementary information

**Additional file 1: Figure S1.** Animated GIF of heat maps of amplicon reads from minutes 1 through 9 of the ONT sequencing runs. Fastq file time slices produced by custom bash script nanotimeparse (available on github: https://github.com/raplayer/nanotimeparse.git).

**Additional file 2: Table S1.** Detailed PCR assay information with in silico analysis. **Table S2**. Summary of singleplex real-time PCR results for individual agents. **Figure S2** Metagenome analyses of reads generated from three different matrix types. Pie chart of representing percentage of reads identified in each sample from individually spiked samples. Percentage of reads that mapped to an amplicon reference (amplicon_mapped), percentage of resulting unmapped reads that then mapped to a set of reference genomes of all tested organisms (contig_mapped), and percentage of resulting unmapped reads that were either classified (classified) or unclassified (unclassified) based on metagenomics classifications using the Kraken (v1) RefSeq database (used for all subsequent Kraken classifications). **Figure S3** Metagenome analyses and comparison of reads generated from PCR amplified and unamplified samples of three different matrix types. Pie chart of percentage of reads identified in mixed cocktail sample backgrounds. Percentage of reads that mapped to an amplicon reference (amplicon_mapped), percentage of resulting unmapped reads that then mapped to a set of reference genomes of all tested organisms (contig_mapped), and percentage of resulting unmapped reads that were either classified (classified) or unclassified (unclassified) based on metagenomics classifications using the Kraken (v1) RefSeq database (used for all subsequent Kraken classifications).

## Data Availability

The datasets generated and/or analyzed during the current study are not publicly available due to the classified nature of the amplicon targets. The custom script for parsing ONT reads by time period is available on GitHub (https://github.com/raplayer/nanotimeparse).

## Abbreviations

AUC: Area Under the Curve
bp: base pairs
CB: Cocktail Buffer
CF: Clean Filter
COTS: Commercial Off the Shelf
DF: Dirty Filter
DoD: Department of Defense
FDA: Food and Drug Administration
FPR: False Positive Rate
gi: gamma-irradiated
LoD: Limit of Detection
MAPQ: Mapping Quality
NGS: Next Generation Sequencing
ONT: Oxford Nanopore Technologies
PCR: Polymerase Chain Reaction
PR: Precision-Recall
PTFE: Polytetrafluoroethylene
QC: Adapter and Quality trimming of sequence reads
ROC: Receiver Operating Characteristic
TGS: Third Generation Sequencing
