# Supplementary figures and images for: Comparison of the performance of an amplicon sequencing assay based on Oxford Nanopore technology to real-time PCR assays for detecting bacterial biodefense pathogens
Source: BMC Genomics. 2020 Feb 17;21:166. doi: 10.1186/s12864-020-6557-5 (PMC7026984; doi:10.1186/s12864-020-6557-5)

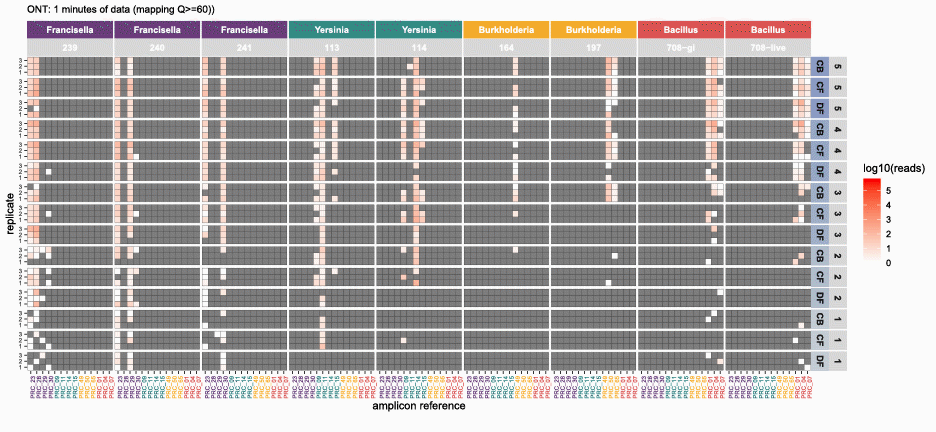

Supplement: Supplementary file 1 — Additional file 1: Figure S1. Animated GIF of heat maps of amplicon reads from minutes 1 through 9 of the ONT sequencing runs. Fastq file time slices produced by custom bash script nanotimeparse (available on github: https://github.com/raplayer/nanotimeparse.git). [file 12864_2020_6557_MOESM1_ESM.gif]
