# Supplementary material for: Comparison of the performance of an amplicon sequencing assay based on Oxford Nanopore technology to real-time PCR assays for detecting bacterial biodefense pathogens
Source: BMC Genomics. 2020 Feb 17;21:166. doi: 10.1186/s12864-020-6557-5 (PMC7026984; doi:10.1186/s12864-020-6557-5)
Supplement: Supplementary file 2 — Additional file 2: Table S1. Detailed PCR assay information with in silico analysis. Table S2. Summary of singleplex real-time PCR results for individual agents. Figure S2 Metagenome analyses of reads generated from three different matrix types. Pie chart of representing percentage of reads identified in each sample from individually spiked samples. Percentage of reads that mapped to an amplicon reference (amplicon_mapped), percentage of resulting unmapped reads that then mapped to a set of reference genomes of all tested organisms (contig_mapped), and percentage of resulting unmapped reads that were either classified (classified) or unclassified (unclassified) based on metagenomics classifications using the Kraken (v1) RefSeq database (used for all subsequent Kraken classifications). Figure S3 Metagenome analyses and comparison of reads generated from PCR amplified and unamplified samples of three different matrix types. Pie chart of percentage of reads identified in mixed cocktail sample backgrounds. Percentage of reads that mapped to an amplicon reference (amplicon_mapped), percentage of resulting unmapped reads that then mapped to a set of reference genomes of all tested organisms (contig_mapped), and percentage of resulting unmapped reads that were either classified (classified) or unclassified (unclassified) based on metagenomics classifications using the Kraken (v1) RefSeq database (used for all subsequent Kraken classifications). [file 12864_2020_6557_MOESM2_ESM.pdf]

Additional Files

Table S1. Detailed PCR assay information with *in silico* analysis.

Organism	PCR Assay ID_Number	Gene	Target Replicon	GenBank Accession Number*	*In Silico* Results TP	TN	FP	FN
B. anthracis	PRC_01	*pagA*	*pXO1*	*CP007665.1*	60	8	19	1
B. anthracis	PRC_04	*pgsB*	*pXO2*	*CP007664.1*	43	13	7	0
B. anthracis	PRC_07	*sasP-B*	*Chr*	*CP007666.1*	60	240	1	2
Y. pestis	PRC_09	*caf1*	*pMT1*	*CP045259.1*	54	0	4	0
Y. pestis	PRC_11	*pst*	*pPCP1*	*CP045262.1*	36	0	3	0
Y. pestis	PRC_14	*lcrV*	*pCD1*	*CP045260.1*	53	1	43	0
Y. pestis	PRC_15	*fadD*	*Chr*	*CP045258.1*	42	541	26	0
F. tularensis	PRC_23	*lpnA*	*Chr*	*CP003049.2*	13	60	52	2
F. tularensis	PRC_28	*Type 1 RMS-S subunit*	*Chr (Type A)*	*CP003049.2*	13	0	3	0
F. tularensis	PRC_29	*IS5 fam Transposase*	*Chr (Type B)*	*CP034466.1*	14	30	6	0
F. tularensis	PRC_30	*KX02_1354*	*Chr (Type N)*	*CP009682.1*	9	38	3	2
B. mallei	PRC_49	*NW99_22070*	*Chr II*	*CP010349.1*	30	0	12	0
B. pseudomallei	PRC_50	*H10_31330*	*Chr II*	*CP025301.3*	119	4	0	0
B. pseudomallei	PRC_65	*H10_06125*	*Chr 1*	*CP025300.3*	115	2	29	0

Counting as positive or negative is contingent upon three features: 1) assay hit - whether or not both primers and the probe match to an organism in at least one database (three NCBI databases were tested Jan 06, 2020: nt, env nt, and gss), 2) amplicon hit - whether or not the amplicon sequence matches an organism in at least one database, and 3) the matched organism. Assay hit implies that amplification is possible in the matched organism and is considered a positive (whether true or false). Additionally, assay hits (primers and probe) must match a database sequence with at least 90% identity and the alignment length must be at least 90% of the primer length. Amplicon hits must match a database sequence with at least 85% identity and the alignment length must be at least 90% of the amplicon length. The following combinations of the three features determine if an alignment is counted as a true or false positive or negative: TP = primer and amplicon hits in the expected organism; TN = no primer hit with or without amplicon hit in any other organism, FP = primer and amplicon hits in any other organism, and FN = no primer hit with or without amplicon hit in the expected organism. *GenBank searched on Jan 03, 2020.

Table S2. Summary of singleplex real-time PCR results for individual agents.

Organism	Strain	***F. tularensis* assays**				***Y. pestis* assays**				***Burkholderia* assays**			***B. anthracis* assays**		
F. tularensis	239	72	85	TN	TN	-	-	-	-	-	-	-	-	-	-
	240	125	TN	FN	TN	-	-	-	-	-	-	-	-	-	-
	241	191	TN	TN	121	-	-	-	-	-	-	-	-	-	-
Y. pestis	113	-	-	-	-	129	110	TN	142	-	-	-	-	-	-
	114	-	-	-	-	111	TN	FN	106	-	-	-	-	-	-
B. mallei	164	-	-	-	-	-	-	-	-	74	TN	TN	-	-	-
B. pseudomallei	197	-	-	-	-	-	-	-	-	TN	106	FN	-	-	-
B. anthracis	708-gi	-	-	-	-	-	-	-	-	-	-	-	58	66	FN
**PCR Assay Number**		**23**	**28**	**29**	**30**	**9**	**11**	**14**	**15**	**49**	**50**	**65**	**01**	**04**	**07**
**Probe Dye / Channel**		**FAM**	**VIC**	**NED**	**CY5**	**FAM**	**VIC**	**NED**	**CY5**	**FAM**	**VIC**	**NED**	**FAM**	**VIC**	**NED**

Values in the cells indicate an observed positive result ($C_t < 40$) where a positive result was expected, and represent a PCR efficiency percentage. A minus sign (-) indicates the assay-organism combination was not tested. Cells containing FN indicate an observed false negative result. Cells containing TN indicate an observed negative or undetected result ($C_t \geq 40$) where a positive result was *not* expected.

Figure S1: Animated GIF of heat maps of amplicon reads from minutes 1 through 9 of the ONT sequencing runs. Fastq file time slices produced by custom bash script nanotimeparse (available on github: https://github.com/raplayer/nanotimeparse.git).

Figure S2. Metagenome analyses of reads generated from three different matrix types

Pie chart of representing percentage of reads identified in each sample from individually spiked samples. Percentage of reads that mapped to an amplicon reference (amplicon_mapped), percentage of resulting unmapped reads that then mapped to a set of reference genomes of all tested organisms (contig_mapped), and percentage of resulting unmapped reads that were either classified (classified) or unclassified (unclassified) based on metagenomics classifications using the Kraken (v1) RefSeq database (used for all subsequent Kraken classifications).

Additional file 3: FigureS3.pdf

Figure S3. Metagenome analyses and comparison of reads generated from PCR amplified and unamplified samples of three different matrix types

Pie chart of percentage of reads identified in mixed cocktail sample backgrounds. Percentage of reads that mapped to an amplicon reference (amplicon_mapped), percentage of resulting unmapped reads that then mapped to a set of reference genomes of all tested organisms (contig_mapped), and percentage of resulting unmapped reads that were either classified (classified) or unclassified (unclassified) based on metagenomics classifications using the Kraken (v1) RefSeq database (used for all subsequent Kraken classifications).

Figure S2.

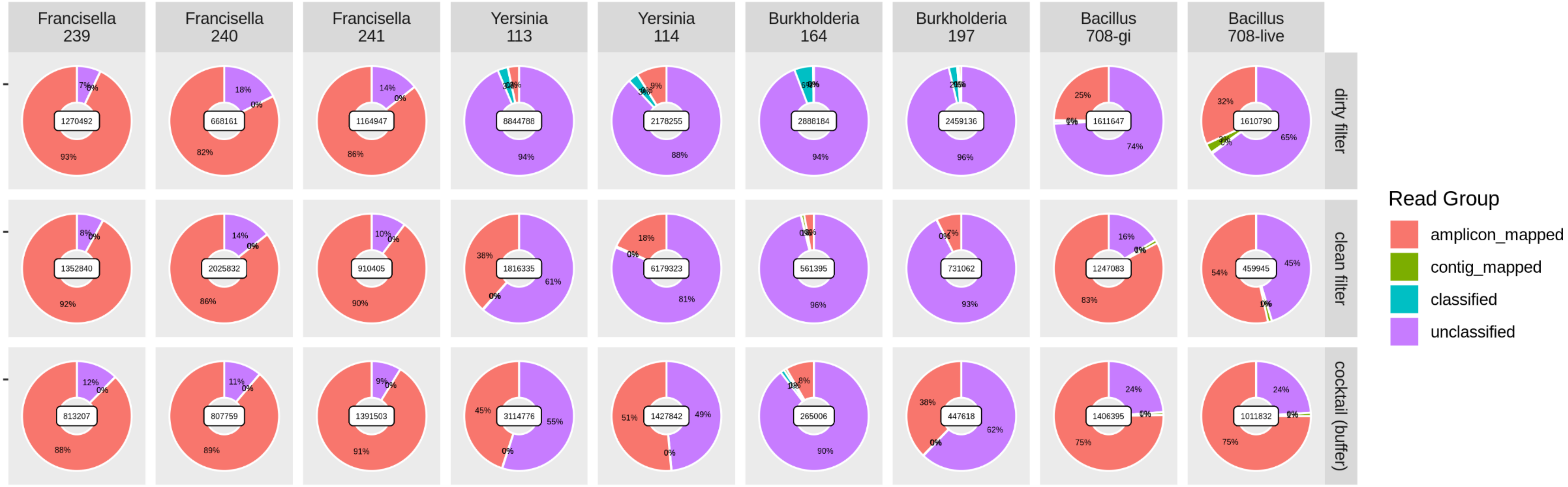

Francisella 239
Francisella 240
Francisella 241
Yersinia 113
Yersinia 114
Burkholderia 164
Burkholderia 197
Bacillus 708-gi
Bacillus 708-live
dirty filter
clean filter
cocktail (buffer)
1270492
668161
1164947
8844788
2178255
2888184
2459136
1611647
1610790
1352840
2025832
910405
1816335
6179323
561395
731062
1247083
459945
813207
807759
1391503
3114776
1427842
265006
447618
1406395
1011832
Read Group
amplicon_mapped
contig_mapped
classified
unclassified

Figure S3.

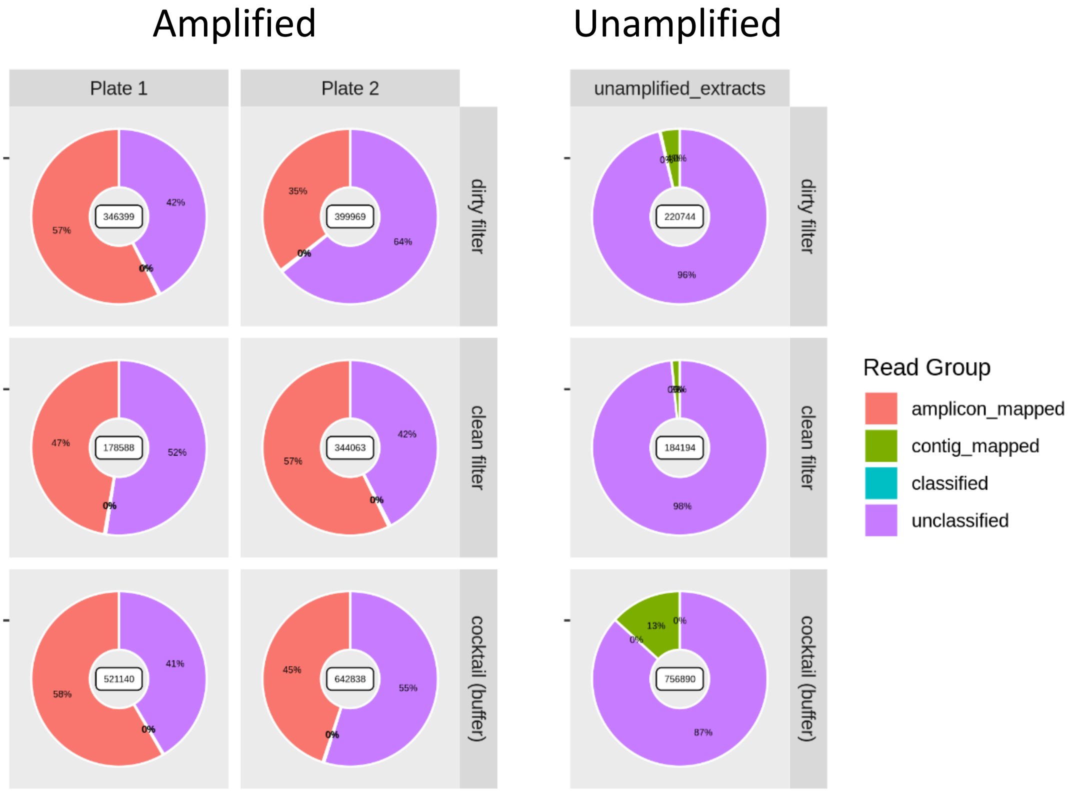
Amplified
Unamplified
Plate 1
Plate 2
unamplified_extracts
dirty filter
clean filter
cocktail (buffer)
346399
57%
42%
0%
399969
35%
64%
0%
220744
96%
178588
47%
52%
0%
344063
57%
42%
0%
184194
98%
521140
58%
41%
0%
642838
45%
55%
0%
756890
13%
0%
0%
87%
Read Group
amplicon_mapped
contig_mapped
classified
unclassified
